# A high-concentrate diet induces inflammatory injury via regulating Ca^2+^/CaMKKβ-mediated autophagy in mammary gland tissue of dairy cows

**DOI:** 10.3389/fimmu.2023.1186170

**Published:** 2023-05-01

**Authors:** Meijuan Meng, Xuerui Li, Zihan Wang, Ran Huo, Nana Ma, Guangjun Chang, Xiangzhen Shen

**Affiliations:** College of Veterinary Medicine, Nanjing Agricultural University, Nanjing, Jiangsu, China

**Keywords:** subacute rumen acidosis, mammary gland tissue, CaMKKβ, autophagy, inflammation, LPS, bovine mammary epithelial cells

## Abstract

**Introduction:**

Calmodulin-dependent protein kinase β (CaMKKβ) is closely related to Ca^2+^ concentration. An increase in Ca^2+^ concentration in the cytoplasm activates CaMKKβ, and activated CaMKKβ affects the activities of AMPK and mTOR and induces autophagy. A high-concentrate diet leads to Ca^2+^ disorder in mammary gland tissue.

**Objectives:**

Therefore, this study mainly investigated the induction of mammary gland tissue autophagy by a high-concentrate diet and the specific mechanism of lipopolysaccharide (LPS)-induced autophagy in bovine mammary epithelial cells (BMECs).

**Material and Methods:**

Twelve mid-lactation Holstein dairy cows were fed with a 40% concentrate diet (LC) and a 60% concentrate diet (HC) for 3 weeks. At the end of the trial, rumen fluid, lacteal vein blood, and mammary gland tissue were collected. The results showed that the HC diet significantly decreased rumen fluid pH, with a pH lower than 5.6 for more than 3 h, indicating successfully induction of subacute rumen acidosis (SARA). The mechanism of LPS-induced autophagy in BMECs was studied in vitro. First, the cells were divided into a Ctrl group and LPS group to study the effects of LPS on the concentration of Ca^2+^ and autophagy in BMECs. Then, cells were pretreated with an AMPK inhibitor (compound C) or CaMKKβ inhibitor (STO-609) to investigate whether the CaMKKβ–AMPK signaling pathway is involved in LPS-induced BMEC autophagy.

**Results:**

The HC diet increased the concentration of Ca^2+^ in mammary gland tissue and pro-inflammatory factors in plasma. The HC diet also significantly increased the expression of CaMKKβ, AMPK, and autophagy-related proteins, resulting in mammary gland tissue injury. In vitro cell experiments showed that LPS increased intracellular Ca^2+^ concentration and upregulated protein expression of CaMKKβ, AMPK, and autophagy-related proteins. Compound C pretreatment decreased the expression of proteins related to autophagy and inflammation. In addition, STO-609 pretreatment not only reversed LPS-induced BMECs autophagy but also inhibited the protein expression of AMPK, thereby alleviating the inflammatory response in BMECs. These results suggest that inhibition of the Ca^2+^/CaMKKβ–AMPK signaling pathway reduces LPS-induced autophagy, thereby alleviating inflammatory injury of BMECs.

**Conclusion:**

Therefore, SARA may increase the expression of CaMKKβ by increasing Ca^2+^ levels and activate autophagy through the AMPK signaling pathway, thereby inducing inflammatory injury in mammary gland tissue of dairy cows.

## 1 Introduction

With increasing demand for milk and dairy products, feeding high-concentrate (HC) diets to dairy cows to rapidly improve lactation performance has become the norm. Although feeding an HC diet can improve economic efficiency in the short term, long-term feeding can lead to a series of nutritional metabolic diseases, the most important of which is subacute rumen acidosis (SARA) (1), defined as a rumen pH of 5.0–5.5 for a duration exceeding 3 h per day (2). When rumen pH remains low for a long time, Gram-negative bacteria in rumen lyse and release large amounts of lipopolysaccharide (LPS) (3), resulting in mammary gland tissue damage in dairy cows (4).

Under normal conditions, autophagy is conducive to maintaining the homeostasis of the intracellular environment and can remove alien and provide energy for cell activities (5). Studies have demonstrated an important role of Ca^2+^ in autophagy. Elevation of intracellular Ca^2+^ activates multiple autophagy signaling kinases and proteasomes to induce autophagy (6–8). Calmodulin-dependent protein kinase β (CaMKKβ) is a classical downstream molecule of Ca^2+^ signaling, the activity of which changes with Ca^2+^ concentration. 5’-AMP-activated protein kinase (AMPK) is an important energy receptor in cells with an important role in autophagy (9, 10). Increased intracellular Ca^2+^ concentration leads to phosphorylation of AMPK (11, 12). Activation of AMPK can inhibit the activity of mechanistic target of rapamycin (mTOR), which, in turn, activates downstream ULK1 and ultimately leads to autophagy (9). In addition, AMPK can directly phosphorylate the downstream ULK1 to induce autophagy (13, 14). Studies have shown that inhibition of CaMKKβ results in a decrease in AMPK phosphorylation (15). Increased intracellular Ca^2+^ activates the CaMKKβ/AMPK/mTOR pathway and enhances autophagy, whereas decreased Ca^2+^ concentration inhibits autophagy (16, 17). Therefore, Ca^2+^ and CaMKKβ have important roles in autophagy. It has been reported that cinacalcet alleviates apoptosis and oxidative stress by activating CaMKKβ–LKB1–AMPK signaling in glomerular endothelial cells and podocyte in the kidney *via* increasing intracellular Ca^2+^ concentration (18). Research also indicates that Trpc5 induces autophagy of cancer cells through the CaMKKβ/AMPKα/mTOR pathway, which promotes drug resistance in breast cancer (19). These findings raise the question of whether there is a relationship between the Ca^2+^ homeostasis imbalance induced by HC diets and autophagy. The purpose of the present study was to study the status of mammary gland tissue autophagy in cows with SARA induced by an HC diet and the specific mechanism of LPS-induced autophagy in bovine mammary epithelial cells (BMECs).

We hypothesized that the HC diet would induce autophagy by activating the CaMKKβ–AMPK signaling pathway, leading to inflammatory injury of mammary gland tissue in dairy cows. This hypothesis was tested by measuring the expression of autophagy-related proteins in mammary gland tissue, and the mechanism was verified using CaMKKβ and AMPK inhibitors *in vitro*. The results provide new insight into how mammary gland tissue could be protected against the negative effects of LPS in dairy cows and indicate a new target for the treatment and prevention of dairy mastitis.

## 2 Materials and methods

### 2.1 Ethics statement

The experimental procedures were carried out in accordance with the Guidelines for Experimental Animals of the Ministry of Science and Technology (2006, Beijing, China) and approved by the Animal Ethics Committee of Nanjing Agricultural University (approval number: SYXK-2017-0027).

### 2.2 Animal experiment design and diet

Twelve healthy multiparous mid-lactation Holstein cows (parities 2–3, body weight 651 ± 54 kg, lactation days 233 ± 16 days) were randomly divided into two groups, with six replicates per group: one group was fed a diet with 40% concentrate [low concentrate (LC)], and the other group was fed a diet with 60% concentrate (HC). Before the experiment, each cow was fitted with rumen fistulas. All animals were euthanized, and samples of mammary gland tissue were collected after 3 weeks. Experimental diets are shown in **Table 1**.

**Table 1:** Ingredients and nutrient contents of the low-concentrate diet and high-concentrate diet (DM basis, %).

Items	Diet (Content)	
	LC	HC
Ingredients		
Maize	19.40	24.92
Soybean meal	13.50	13.48
Barley	0.00	12.00
Distillers dried grains with soluble	3.80	5.91
silage corn	12.00	6.00
Alfalfa	24.00	17.00
Oat grass	24.00	17.00
Limestone	0.80	1.48
CaHPO_4_	1.10	0.92
NaCl	0.40	0.37
Premix^1)^	1.00	0.92
Total	100.00	100.00
Nutrient levels^2)^		
Crude protein (%)	16.16	16.21
Calcium (%)	1.14	1.18
Phosphorus (%)	0.52	0.51
Neutral detergent fiber (%)	36.14	29.92
Non-fiber carbohydrate (%)	35.39	42.34
Ash (%)	5.97	4.87
Net energy (MJ/kg)	1.57	1.64
Crude fat (%)	3.05	3.05
Starch (%)	17.96	27.82
NFC/NDF	0.99	0.71

^1)^The premix provided the following per kg of diet: vitamin A, 22.5 KIU/kg; vitamin D3, 5.0 KIU/kg; vitamin E, 37.5 IU/kg; vitamin K3, 5.0 mg/kg; Cu, 25.6 mg/kg; Fe, 159.3 mg/kg; Zn, 111.9 mg/kg; and Mn, 63.5 mg/kg.

^2)^ Nutrient levels were estimated values.

### 2.3 Sample collection

The cows were individually housed in a room with solid wooden floor pens. Throughout the experiment, the dairy cows were fed twice at 09:00 and 16:00. Water and food were allowed *ad libitum*. On the last day of the experiment, blood was collected *via* the lacteal vein catheters of the cows and centrifuged at 3,000 rpm for 15 min. Then, plasma was collected and stored at −20°C for the determination of IL-6, TNF-α, and IL-1β levels. At the end of the experiment, all the cows were humanely euthanized (killed with a captive bolt) on the same day. The collected mammary gland was first cut into small pieces, then washed with saline solution to remove as much milk and blood as possible. One part of the mammary gland was stored in a −80°C freezer for the determination of genes and proteins, and the other part was fixed with 4% paraformaldehyde for histochemical analysis of LC3, CaMKKβ, and p62.

### 2.4 Reagents

LPS from *Escherichia coli* 055:B5 was purchased from Sigma (L2880, Sigma-Aldrich, St. Louis, MO, USA). STO-609, a specific CaMKKβ inhibitor, was purchased from Shanghai Yuanye Bio-Technology Co., Ltd., Shanghai, China (S80770). Compound C, an AMPK inhibitor, was purchased from MedChemExpress, State of New Jersey, USA (HY-13418A).

### 2.5 Cell experimental design and treatment

LPS is usually used to simulate SARA induction in *in vitro* models. To verify whether the CaMKKβ–AMPK signaling pathway is involved in mammary gland tissue autophagy induced by an HC diet in dairy cows, LPS stimulation of BMECs was used here as a model, as our research group has used BMECs as an *in vitro* model in many previous studies (20, 21). We studied the mechanism through cell experiments *in vitro* as follows.

Immortalized BMECs obtained from mammary gland tissue of a mid-lactation Holstein dairy cow were purchased from Shanghai Tongpai Biotechnology Co. Ltd. (Shanghai, China) and established and characterized by Zhao et al. (22). BMECs were cultivated, resuscitated, and passaged in complete medium (90% RPMI 1640 + 10% fetal bovine serum + 5% penicillin–streptomycin), and some cells were frozen for subsequent tests. Cells from passages 4–7 were used in this assay.

(1) LPS concentration gradient treatment. To study the effects of different concentrations of LPS on autophagy-related genes and proteins in BMECs, the cells were treated with different concentrations of LPS (2, 4, 8, 16, and 20 μg/ml) for 3 h. The mRNA and protein expression of ATG5, p62, and LC3 and the fluorescence intensity of p62 and LC3 were determined to identify the optimal concentration of LPS.
(2) AMPK inhibitor (compound C) + LPS treatment. Cells were divided into a Ctrl group (Ctrl), an LPS group (4 μg/ml LPS, 3 h), a compound C pretreatment group (CLPS; pretreatment for 12 h with 5 μM compound C, followed by stimulation with 4 μg/ml LPS for 3 h), and a compound C treatment group (cells treated with 5 μM compound C for 12 h).
(3) CaMKKβ inhibitor (STO-609) + LPS treatment. Cells were divided into a Ctrl group, an LPS group (treatment with 4 μg/ml LPS for 3 h), an STO-609 pretreatment group (pretreatment with 4 μM STO-609 for 12 h, followed by stimulation with 4 μg/ml LPS for 3 h), and an STO-609 treatment group (treatment with 4 μM STO-609 for 12 h).

### 2.6 Rumen pH, Ca^2+^, and inflammatory cytokine measurements

Rumen fluid was sampled through the rumen fistula at 1, 2, 3, 4, 5, and 6 h after feeding in the morning on days 7, 14, and 21 of the trial period. Rumen fluid was filtered with four layers of gauze. The rumen pH was measured using a pH meter (HANNA Instruments, Romania).

Lacteal vein plasma was removed from storage in a refrigerator at −20°C, and the inflammatory cytokine IL-1β (CK-EN77027), IL-6 (CK-EN77030), and TNF-α (CK-EN77163) contents were detected by enzyme-linked immunosorbent assay according to the kit instructions. The calcium content in the mammary gland of dairy cows was determined using a Calcium Colorimetric Assay Kit (S1063S, Beyotime) according to the instructions.

### 2.7 Histopathological examination of mammary gland tissue

Fixed mammary tissue was dehydrated and embedded in paraffin. Then, tissues were cut into 5-μm sections using a microtome and stained with hematoxylin–eosin (H&E), following the method described by Wang et al. (23). Finally, histopathological changes were observed and recorded using a light microscope.

### 2.8 Immunohistochemical analysis

Immunohistochemical analysis was essentially as described in our previous study (24). Prepared sections were incubated with specific antibodies (against LC3, CaMKKβ, and p62) at 4°C overnight. After washing with phosphate-buffered saline (PBS), slices were incubated with the corresponding secondary antibody at room temperature for 50 min. The slices were then stained with diaminobenzidine (DAB) chromogenic solution and redyed with hematoxylin. Finally, the expression of target proteins LC3, p62, and CaMKKβ ware observed by an optical microscope, and photographs were taken.

### 2.9 Cell viability

A cell counting Kit-8 (CCK-8) kit was used to detect cell viability. BMECs were inoculated in 96-well plates overnight and treated with LPS, compound C, and STO-609 at different concentrations for 12 h. Then, 10 μl of CCK-8 solution was added to each well, and the plates were placed in an incubator for 4 h. Cell viability was calculated by reading the optical density value at 450 nm with a microplate reader.

### 2.10 Immunofluorescence

Cells were inoculated in a 24-well plate with sterile slides and cultured overnight before being treated according to the experimental design. After treatment, the cells were washed with PBS three times, and fixed with 4% paraformaldehyde at room temperature for 15 min. Then, they were permeated with 0.3% Triton X-100 at room temperature for 15 min, and blocked with a solution of 5% bovine serum albumin for 30 min. Next, p62 CaMKKβ and LC3 antibodies were added, followed by incubation at 4°C overnight. On the second day, the corresponding secondary antibodies were added according to the selection of primary antibodies, followed by incubation for 1 h at 37°C. Nuclei were stained with 4',6-diamidino-2-phenylindole (DAPI) for 5 min. The slides were removed, and cells were fixed with an anti-quenching agent. The fluorescence status of LC3 and p62 was observed under a laser confocal microscope, following the method as described by Lian et al. (25).

### 2.11 RNA extraction and quantitative real-time PCR

mRNA was isolated from the mammary gland tissue and cell samples using a FastPure Cell/Tissue Total RNA Isolation Kit V2 (RC112, Vazyme, Nanjing, China) according to the manufacturer’s instructions. The RNA was reverse-transcribed into cDNA using a cDNA reverse-transcription kit [11120ES60, Yeasen Biotechnology (Shanghai) Co., Ltd.], again according to the manufacturer’s instructions. Quantitative RT-PCR was performed with a SYBR Green Premix Pro TaqHS qPCR Kit [AG11701, Accurate Biotechnology (Hunan) Co., Ltd., Changsha] using an Applied Biosystems 7500 RT-PCR system (Life Technologies, CA, USA). Glyceraldehyde phosphate dehydrogenase (GAPDH) was used as an internal reference gene for the normalization of gene expression. The primers in the experiment were designed and assessed with Oligo 7.0 (Molecular Biology Insights, Inc., Colorado Springs, CO, USA). The specific primers were synthesized by Generay Biotechnology (Shanghai, China), according to the sequences shown in **Table 2**. The 2^−ΔΔCt^ method was used for relative quantification.

**Table 2:** Primers used in quantitative real-time PCR analysis.

Gene	Forward primer	Reverse primer	Size (bp)	GenBank accession
ULK1	CGCCGTCAAGTGCATTAACAAG	TCGGACAAAGCCACGATGTT	111	NM_003565.4
Beclin1	GAACCTCAGCCGAAGACTAAA	CTGACACACAGAGCTCACCT	107	NM_001033627.2
ATG10	GTCAGTGAGGGACACACTTTAG	AGGAGCAGGTACCTCAGAAT	105	NM_001083531.1
ATG5	CACTAGGGCTGGTCTTACTTTG	GACTTGCAGTGGTCCTGTAAA	100	NM_001034579.2
ATG16L1	CGTACCAAACAGGCATGAGATA	GCAGTTCAGTCAGCTCTTCTT	113	NM_001191389.2
ATG7	CGGTTGCCGGAAGTTGGG	GCTTCGTCTAGCCGGTACTC	164	NM_001142967.1
ATG12	TCGACCAGCTGGCTTTATTAC	GTGCTCTCTTGGCTAGATGTT	145	NM_001076982.1
SQSTM1	CAGCACAGAGGAGAAGTGTAG	GACTCCAGGGCGATCTTATTC	126	NM_176641.1
ATG4A	CCGTGGTCATCGAAGATATCAA	TTGGTTGGAAGCAGTCAGAG	101	NM_001001171.1
ATG3	GTGAAGGCTTACCTACCATCAG	CCCATCGCCATCATCTTCTT	129	NM_001075364.1
MAP1LC3A	CTGTAAAGAGGTGCAGCAGAT	ACCAGGAACTTGGTCTTGTC	114	NM_001046175.1
ATG14	ACAACGGCACCAGGAAAAGA	CACGTCAGCAGGATCTCTCG	199	NM_001192099.1
CaMKK2	CCTGTCTGAGCCCAAGGAAG	CGCTACTCCATCACCTCGTC	183	NM_001075390.1
GAPDH	GGGTCATCATCTCTGCACCT	GGTCATAAGTCCCTCCACGA	176	NM_001034034.2

### 2.12 Protein extraction and Western blotting

Total protein of mammary gland tissue (100 mg) and cell samples were extracted using radio Immunoprecipitation Assay (RIPA) lysis buffer. Protein concentrations were determined with a Bicinchoninic Acid (BCA) kit, and proteins were diluted to 4 μg/μl. A 10-μl protein sample was separated on polyacrylamide gel electrophoresis gels of different concentrations; then, the separated proteins were transferred to nitrocellulose. Membranes were incubated in 7.5% milk for 1 h and finally incubated overnight with primary antibody at 4°C (**Table 3**). The strips were washed and incubated with specific secondary antibodies for 2 h. The protein bands were washed again and observed with a Bio-Rad Molecular Imager^®^ 200 ChemiDoc™ XRS + Imaging System (Bio-Rad, Berkeley). GAPDH and β-actin were used to normalize protein expression. The final result for each target protein is presented as relative abundance to reference protein.

**Table 3:** Antibody information for Western blot determination.

Name of antibody	Dilution ratio	Article number	Manufacturers	
LC3	1:1000	14600-1-AP	Proteintech	
IL-1β	1:500	AF5103	Affnity
TNF-α	1:1000	AF8208	Beyotime
SQSTM1/p62	1:500	AF5384	Affnity	
Beclin1	1:500	AF5128	Affnity	
ATG14	1:500	AF7912	Affnity	
ULK1	1:500	AF8307	Beyotime	
CaMKKβ	1:500	11549-I-AP	Proteintech	
LAMP2α	1:1000	AF1036	Beyotime	
ATG5	1:500	A0203	ABclonal	
AMPK	1:1000	AF1627	Beyotime	
p-AMPK	1:1000	AF2677	Beyotime	
mTOR	1:1000	AF1648	Beyotime	
p-mTOR	1:1000	AF5869	Beyotime	
ATG16L	1:500	AF6252	Beyotime	
GAPDH	1:5000	60004-1-Ig	Proteintech	
Actin	1:1000	AA128	Beyotime	

### 2.13 Statistical analysis

SPSS 20.0 was used for statistical analysis (SPSS Inc., Chicago, IL), and data are expressed as the mean and standard error of the mean (mean ± SEM). All experiments were performed in triplicate. Rumen fluid pH was analyzed using a mixed model in SAS 9.2 (SAS Institute Inc.); *t*-tests were used to analyze the differences between the HC diet and LC diet groups, and between the Ctrl and LPS groups in the cell experiments, and data for other cell experiments were analyzed using one-way analysis of variance with Dunnett’s post-test in SPSS 20.0 Statistics for Windows (IBM Corp., New York, NY, USA). *p* < 0.05 was considered to indicate a significant difference, *p* < 0.01 an extremely significant difference, and *p* > 0.05 no significant difference.

## 3 Results

### 3.1 Rumen pH, inflammatory factors, Ca^2+^ content, and H&E staining

**Figure 1:**
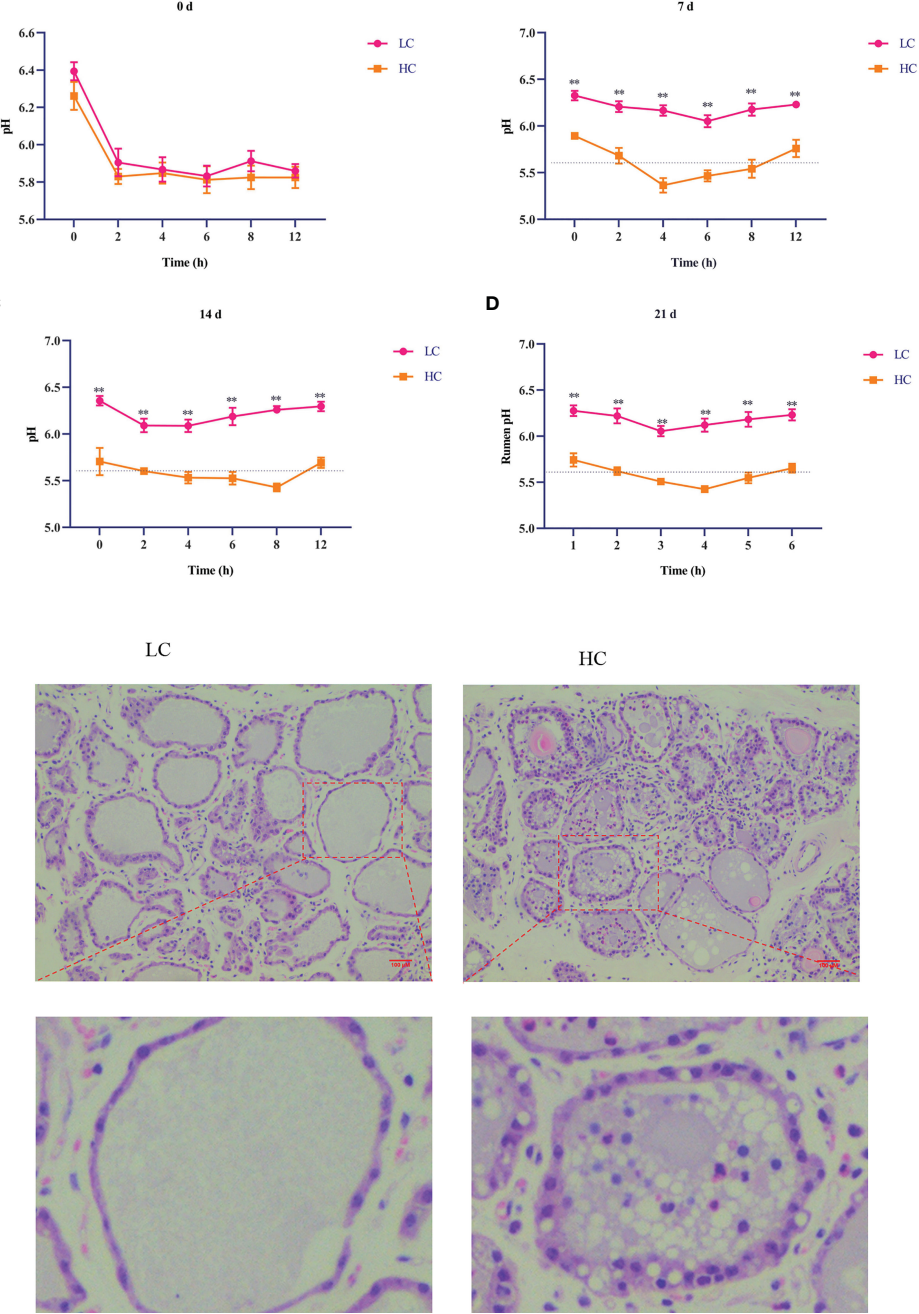
Effects of high-concentrate (HC) diet on rumen pH and hematoxylin and eosin (H&E) staining of mammary gland tissue of dairy cows. Rumen pH of dairy cows at different times **(A–D)**. All results are expressed as the mean ± SEM. ^**^ *p* < 0.01. H&E staining was performed on mammary tissue sections (100× magnification) of dairy cows fed low-concentrate (LC) and HC diets **(E)**. Red bar, 100 μm.

As shown in **Figure 1**, rumen pH in the HC group was lower than that in the LC group on days 7, 14, and 21 (*p* < 0.01, **Figures 1A–D**). In the HC group, rumen pH was less than 5.6 for more than 3 h in a day, indicating successful induction of SARA. As shown in **Table 4**, compared with the LC group, the HC group showed significantly increased expression of inflammatory factors IL-6, TNF-α, and IL-1β in the lacteal vein (*p* < 0.01) and increased Ca^2+^ content in the mammary gland tissue of dairy cows (*p* < 0.01). H&E staining showed that the acinar structure of the mammary gland tissue of dairy cows in the LC group was intact, without tissue damage or inflammatory cell infiltration, whereas the acinar structure of the mammary gland tissue of dairy cows in the HC group was severely damaged, with partial disappearance of the acinar contour and obvious inflammatory cell infiltration (**Figure 1E**).

**Table 4:** Effects of different diets on the concentration of Ca^2+^ in mammary tissue and proinflammatory cytokine content in the plasma of dairy cows.

Items	Diet		*p*-value
	LC	HC	
Ca (μg/g)	40.26 ± 5.36	50.88 ± 5.95	<0.01
IL-6 (pg/ml)	114.12 ± 8.65	154.74 ± 10.76	<0.01
IL-1β (pg/ml)	365.2 ± 51.89	495.24 ± 20.15	<0.01
TNF-α (pg/ml)	102.83 ± 15.54	154.45 ± 17.53	<0.01

### 3.2 Expression of autophagy-related genes and proteins in mammary gland tissue

**Figure 2:**
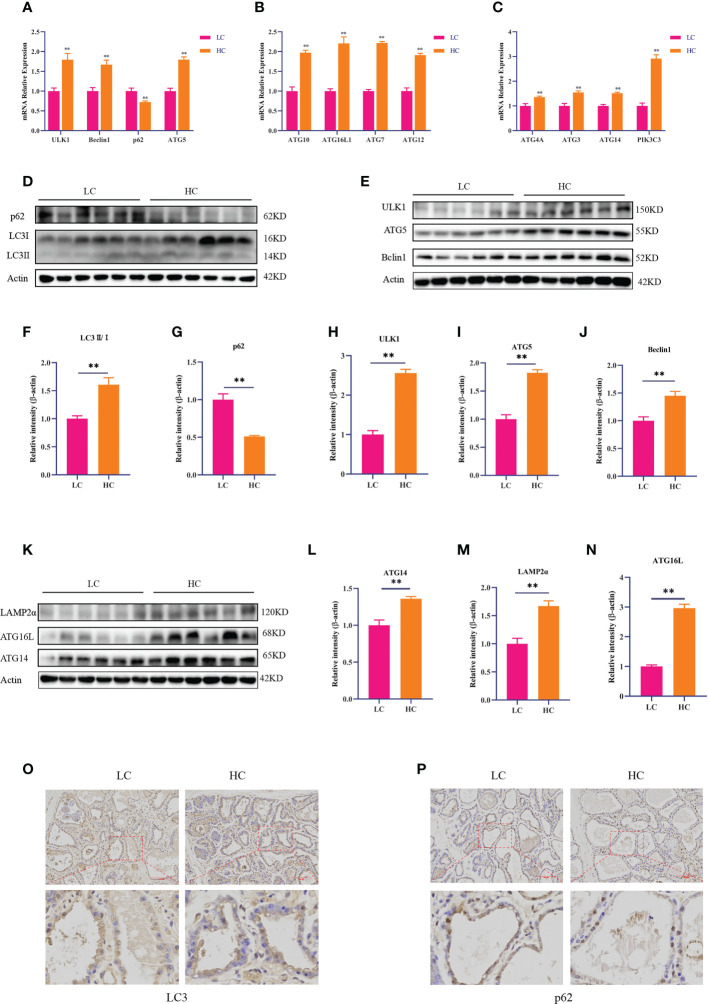
Expression of autophagy-related genes and proteins in mammary gland tissue of dairy cows fed low-concentrate (LC) and high-concentrate (HC) diets. mRNA expression of autophagy-related genes **(A–C)**. Protein expression levels of LC3, p62, ULK1, Beclin1, ATG5, ATG14, ATG16L, and LAMP2α **(D, E, K)**. Protein abundance was normalized to the respective abundance of β-actin **(F–J, L–N)**. Sections of mammary gland tissue from dairy cows in the LC diet and HC diet groups, immunostained for LC3 and p62 (200× magnification, **(O, P)**. Red bar, 200 μm. Results are presented as the mean ± SEM. ^**^ *p* < 0.01.

RT-PCR and Western blotting were used to detect the expression of autophagy-related genes and proteins. The HC diet significantly upregulated the mRNA expression of ULK1, Beclin1, ATG3, ATG4, ATG4A, ATG5, ATG7, ATG10, ATG8, ATG12, and ATG16L compared with the LC diet (*p* < 0.01). However, the mRNA expression of p62 was significantly decreased in the HC group (**Figures 2A–C**, *p* < 0.01). Compared with the LC diet, the HC diet increased the expression of autophagy-related proteins ULK1, ATG5, Beclin1, ATG14, LAMP2α, LC3II/I, and ATG16L (*p* < 0.01), whereas it significantly decreased the expression of p62 (*p* < 0.01, **Figures 2D–N**).

The immunohistochemical DAB staining depth of LC3 in the HC group was higher than that in the LC group. However, the DAB staining depth of p62 in the HC group was lower than that in the LC group (**Figures 2O, P**).

### 3.3 CaMKKβ/AMPK/mTOR signaling pathway in mammary gland tissue

**Figure 3:**
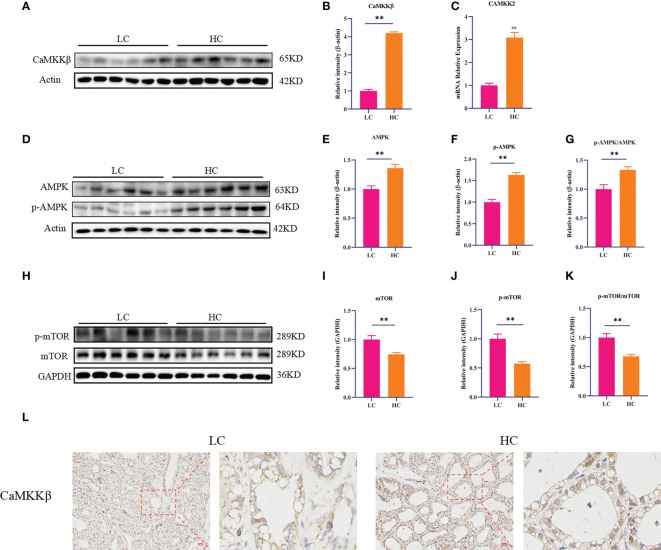
CaMKKβ/AMPK/mTOR signaling pathway in mammary gland tissue of dairy cows. The mRNA abundance of CaMKK2 **(C)**. Protein levels of CaMKKβ, AMPK, p-AMPK, mTOR, p-mTOR, and p62 **(A, B, H)**. Protein expression was normalized to the respective abundance of β-actin and GAPDH **(C–G, I–K)**. Sections of dairy cows’ mammary gland tissue immunostained for CaMKKβ in the LC diet and HC diet (200× magnification, **(L)**. Red bar, 100 μm. All results are expressed as the mean ± SEM. ^**^ *p* < 0.01.

CaMKKβ is a calcium-dependent serine/threonine protein kinase whose activity is closely related to Ca^2+^ concentration. To determine whether an HC diet could activate CaMKKβ by inducing a Ca^2+^ homeostasis imbalance, we first studied the effect of the HC diet on CaMKKβ by RT-PCR and Western blotting. Compared with the LC group, the HC group showed elevated mRNA expression of CaMKK2 and protein expression of CaMKKβ in mammary gland tissue of dairy cows (**Figures 3A–C**, *p* < 0.01). CaMKKβ specifically regulates AMPK and performs a variety of important biological functions when activated by intracellular Ca^2+^. Subsequently, we determined the protein expression of AMPK and mTOR downstream of CaMKKβ. The protein levels of p-AMPK and AMPK and the ratio of p-AMPK to AMPK in the HC group were higher than those in the LC group (*p* < 0.01). By contrast, the protein expression of p-mTOR and mTOR, and the ratio of p-mTOR to mTOR were lower in the HC group (**Figures 3D–K**, *p* < 0.01). Immunohistochemical staining also showed that the HC diet upregulated protein expression of CaMKKβ (**Figure 3L**).

### 3.4 LPS induces autophagy in BMECs

**Figure 4:**
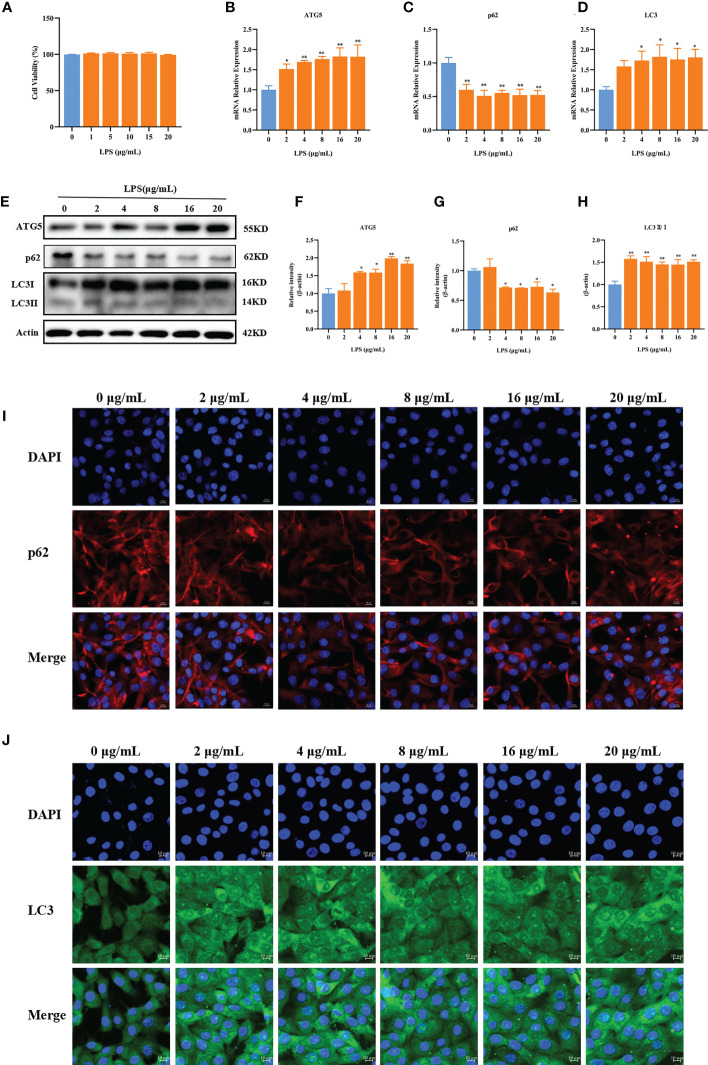
LPS can induce autophagy. Effects of LPS on the cell viability of BMECs **(A)**. Effects of different concentrations of LPS on the mRNA expression of p62, ATG5, and LC3 in BMECs for 3 h **(B–D)**. Effects of different concentrations of LPS on the protein expression of p62, ATG5, and LC3II/I in BMECs for 3 h **(E–H)**. Immunofluorescence results of p62 and LC3 in BMECs treated with different concentration LPS in BMECs for 3 h **(I, J)**. Scale bar, 10 μm. ^*^ *p* < 0.05, ^**^ *p* < 0.01, representing significant difference compared with the Ctrl group.

Our previous studies showed that LPS could cause inflammatory damage to BMECs. For instance, 2–20 μg/ml LPS could upregulate the expression of inflammatory factors in BMECs (26, 27). Therefore, we next investigated whether LPS could induce autophagy in BMECs. The results showed that 0–20 μg/ml LPS did not affect cell viability (*p* > 0.05, **Figure 4A**). Compared with LPS at 0 μg/ml, LPS at 2 μg/ml significantly increased the mRNA expression of ATG5 (*p* = 0.039). LPS at 4, 8, 16, and 20 μg/ml significantly increased the mRNA expression of ATG5 (*p* < 0.01). The mRNA expression of p62 was decreased after LPS treatment at all doses (*p* < 0.01, **Figures 4B, C**). LPS at 4, 8, 16, and 20 μg/ml significantly increased mRNA expression of LC3 (*p* = 0.030, *p* = 0.016, *p* = 0.025, and *p* = 0.018, respectively, **Figure 4D**) and protein expression of ATG5 (*p* = 0.01, *p* = 0.01, *p* < 0.01, and *p* < 0.01, respectively), whereas it decreased protein expression of p62 (*p* = 0.030, *p* = 0.27, *p* = 0.035, and *p* = 0.010, respectively). Compared with 0 μg/ml LPS, the protein expression of LC3II/I was significantly increased by LPS at different concentrations (*p* < 0.01, **Figures 4E–H**). Immunofluorescence results showed that different concentrations of LPS increased the fluorescence intensity of LC3 and reduced the fluorescence intensity of p62 (**Figures 4I, J**). These results suggest that LPS can induce autophagy in BMECs.

### 3.5 AMPK is involved in LPS-induced autophagy in BMECs

**Figure 5:**
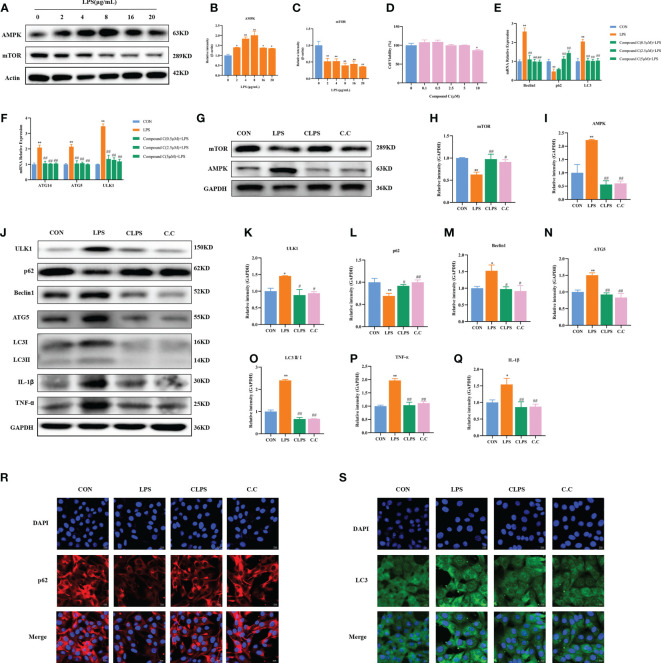
Effect of compound C pretreatment on LPS-induced autophagy in BMECs. Western blot representative bands and quantitative result of AMPK and mTOR protein in BMECs treated with different concentrations of LPS for 3 h **(A–C)**. Effects of compound C on the cell viability of BMECs **(D)**. Effects of compound C pretreatment with different concentrations on LPS-induced autophagy-related genes in BMECs **(E, F)**. Effects of compound C pretreatment on AMPK and mTOR proteins **(G–I)**. Western blot representative bands and quantitative result of autophagy and inflammation proteins in BMECs treated with LPS after compound C pretreatment **(J–Q)**. Immunofluorescence results of p62 and LC3 in BMECs treated with LPS after compound C pretreatment **(R, S)**. Scale bar, 10 μm. ^*^ *p* < 0.05, ^**^ *p* < 0.01, representing significant difference compared with Ctrl group; ^#^ *p* < 0.05 and ^##^ *p* < 0.01, representing significant difference compared with LPS.

Compared with 0 μg/ml LPS, LPS at 2 μg/ml (*p* = 0.025), 4 μg/ml (*p* < 0.01), 8 μg/ml (*p* < 0.01), 16 μg/ml (*p* = 0.029), and 20 μg/ml (*p* = 0.039) significantly increased the protein expression of AMPK (**Figures 5A, B**). Compared with 0 μg/ml LPS, various concentrations of LPS significantly reduced the protein expression of mTOR (*p* < 0.01, **Figures 5A–C**).

To confirm the role of AMPK in LPS-induced autophagy of BMECs, the cells were treated with compound C (AMPK inhibitor) at different concentrations for 12 h and then treated with 4 μg/ml LPS for 3 h. The CCK-8 assay results showed that 0–5 μM compound C did not affect cell activity (*p* > 0.05), whereas 10 μM compound C significantly decreased cell activity (*p* < 0.01, **Figure 5D**).

Compared with the Ctrl group, the LPS group showed significantly increased mRNA expression of Beclin1, LC3, ATG5, ATG14, and ULK1, as well as reduced mRNA expression of p62 (*p* < 0.01). Compared with LPS only, different concentrations of compound C significantly decreased the mRNA expression of Beclin1, LC3, ATG5, ATG14, and ULK1 (*p* < 0.01). Pretreatment with 2.5 and 5 μM compound C significantly increased the mRNA expression of p62 (*p* < 0.01, **Figures 5E, F**). Compared with the Ctrl group, LPS significantly increased the protein expression of AMPK and decreased the protein expression of mTOR (*p* < 0.01, **Figures 5G–I**); these effects were reversed by the addition of compound C. Compared with the Ctrl group, LPS significantly increased the protein expression of ULK1 (*p* = 0.030), Beclin1 (*p* = 0.020), ATG5 (*p* < 0.01), and LC3II/I (*p* < 0.01). Compound C pretreatment significantly reduced the expression of ULK1 (*p* = 0.014), Beclin1 (*p* = 0.017), ATG5 (*p* < 0.01), and LC3II/I (*p* < 0.01). Compared with the Ctrl group, LPS reduced the protein expression of p62 (*p* < 0.01), an effect that was reversed after compound C pretreatment (*p* = 0.028). In addition, compared with the Ctrl group, LPS significantly increased the protein expression of TNF-α (*p* < 0.01) and IL-1β (*p* < 0.05). However, this phenomenon was reversed by pretreatment with compound C. Compared with the LPS group, compound C pretreatment significantly reduced the protein expression of TNF-α and IL-1β (*p* < 0.01, **Figures 5J–Q**). Furthermore, according to the immunofluorescence results, compound C pretreatment decreased the fluorescence intensity of LC3 and increased the fluorescence intensity of p62 (**Figures 5R, S**). These results suggest that inhibition of AMPK can alleviate LPS-induced autophagy, thereby reducing the inflammatory injury of BMECs induced by LPS. They further confirm that the AMPK signaling pathway is involved in LPS-induced autophagy of BMECs.

### 3.6 CaMKKβ–AMPK is involved in LPS-induced autophagy in BMECs

**Figure 6:**
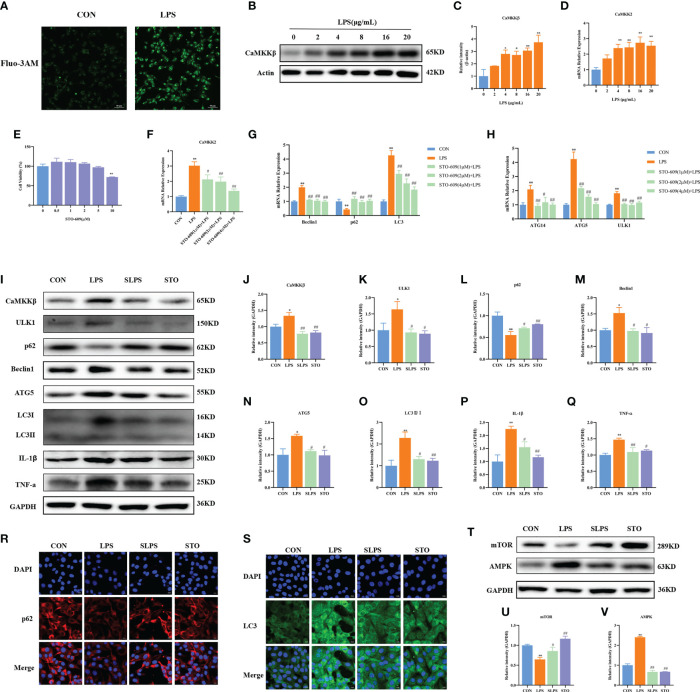
Effect of STO-609 pretreatment on LPS-induced autophagy and inflammation in BMECs. Fluorescence intensity of intracellular Ca^2+^. Scale bar, 50 μm **(A)**. Effects of different concentrations of LPS on the mRNA and protein expression of CaMKKβ in BMECs for 3 h **(B–D)**. Effects of STO-609 on the cell viability of BMECs **(E)**. Effects of STO-609 pretreatment with different concentrations on LPS-induced autophagy-related genes and the mRNA expression of CaMKK2 in BMECs **(F–H)**. WB representative bands and quantitative result of autophagy and inflammation proteins in BMECs treated with LPS after STO-609 pretreatment **(I–Q)**. Immunofluorescence results of p62 and LC3 in BMECs treated with LPS after STO-609 pretreatment. Scale bar, 10 μm **(R, S)**. Effect of STO pretreatment on AMPK and mTOR protein expression in BMECs **(T–V)**. ^*^ *p* < 0.05, ^**^ *p* < 0.01, representing significant difference compared with the Ctrl group; ^#^ *p* < 0.05 and ^##^ *p* < 0.01, representing significant difference compared with LPS.

CaMKKβ activity is usually induced by increased intracellular Ca^2+^ concentration. Therefore, we first determined whether LPS stimulation could upregulate Ca^2+^ levels in BMECs. Compared with the Ctrl group, the LPS group had increased intracellular Ca^2+^ fluorescence intensity (**Figure 6A**). CaMKKβ is one of the classical signaling molecules upstream of AMPK. However, whether CaMKKβ plays an important part in LPS-induced BMEC autophagy through the AMPK–mTOR signaling pathway has yet to be confirmed. Therefore, in this study, we treated cells with different concentrations of LPS. As shown in **Figure 6**, LPS at 4–20 μg/ml significantly upregulated the mRNA expression of CaMKK2 (*p* < 0.01), and LPS at 4 (*p* = 0.01), 8 (*p* = 0.017), 16 (*p* < 0.01), and 20 (*p* < 0.01) μg/ml significantly increased the protein expression of CaMKKβ (**Figures 6B–D**). Therefore, LPS activates CaMKKβ by increasing intracellular Ca^2+^ concentration.

In order to further confirm the role of CaMKKβ in LPS-induced BMECs, STO-609 (a CaMKKβ inhibitor) was used to pretreat BMECs, followed by treatment with 4 μg/ml LPS for 3 h. The results showed that 0–5 μM STO-609 did not affect the activity of BMECs (*p* > 0.05), whereas 10 μM STO-609 significantly decreased their activity (*p* < 0.01, **Figure 6E**). LPS significantly upregulated the mRNA expression of CaMKK2, Beclin1, and LC3 compared with that in the Ctrl group (*p* < 0.01). Pretreatment with 1 μM, 2 μM, and 4 μM STO-609 significantly reduced the mRNA expression of CaMKK2 (*p* = 0.013, *p* < 0.01, and *p* < 0.01, respectively), Beclin1 (*p* < 0.01), ATG5 (*p* < 0.01), ULK1 (*p* < 0.01), ATG14 (*p* < 0.01, *p* = 0.015, and *p* < 0.01, respectively), and LC3 (*p* < 0.01) compared with the LPS group. Compared with the Ctrl group, LPS significantly decreased the mRNA expression of p62 (*p* < 0.01), and STO-609 at 1 μM, 2 μM, and 4 μM significantly increased the mRNA expression of p62 (*p* < 0.01, **Figures 6F–H**). The inhibitory effect of 4 μM STO-609 was the best. Therefore, 4 μM STO-609 was selected for subsequent tests.

Compared with the Ctrl group, LPS significantly upregulated the protein expression of CaMKKβ (*p* = 0.016), an effect that was reversed by the addition of the inhibitor STO-609. Compared with the LPS group, STO-609 pretreatment significantly reduced the protein expression of ULK1 (*p* = 0.022), Beclin1 (*p* = 0.017), LC3II/I (*p* = 0.010), and ATG5 (*p* = 0.030), whereas it significantly increased the protein expression of p62 (*p* = 0.042, **Figures 6I–O**). In addition, compared with the LPS group, STO-609 pretreatment significantly reduced the protein expression of TNF-α (*p* < 0.01) and IL-1β (*p* = 0.024). The immunofluorescence results further showed that compared with the LPS group, STO-609 pretreatment increased the fluorescence intensity of p62 and decreased the fluorescence intensity of LC3 (**Figures 6R, S**). Compared with the Ctrl group, LPS significantly upregulated AMPK protein expression and decreased mTOR protein expression (*p* < 0.01), effects that were reversed by STO-609 pretreatment (*p* < 0.01 and *p* = 0.044, respectively, **Figures 6T–V**). These results indicate that CaMKKβ plays an important part in LPS-induced autophagy of BMECs and suggest that the CaMKKβ–AMPK signaling pathway is involved in LPS-induced BMEC autophagy.

## 4 Discussion

The dairy industry is an important part of animal husbandry and has an important role in the national economy and agricultural development. The demand for dairy products is increasing year by year. Owing to the lack of high-quality roughage in China, dairy cows are usually fed a large amount of concentrate feed in order to improve their production and maximize economic benefit. HC diets contain large amounts of easily decomposed carbohydrates, which can be rapidly digested in the rumen after feeding, resulting in the production of large amounts of volatile fatty acids and lactic acid. When organic acids exceed the absorption and buffering capacity of the rumen, large amounts of organic acids will accumulate in the rumen, leading to an acid–base imbalance in the rumen; this results in a long-term low rumen pH and eventually induces SARA, which damages animal health and production (2). Our results showed that feeding an HC diet significantly reduced rumen pH compared with an LC diet, with a rumen pH of less than 5.6 for more than 3 h a day, indicating a successful model of SARA in dairy cows. Studies have shown that feeding an HC diet can activate inflammatory signaling pathways and induce mammary inflammatory responses (28). In the present study, the HC diet increased the concentration of inflammatory cytokines in the lacteal vein and the content of Ca^2+^ in mammary gland tissue of dairy cows. H&E staining results also showed that feeding an HC diet could damage the mammary gland tissue structure of dairy cows, with infiltration of large numbers of inflammatory cells in acini. These results further demonstrated that feeding an HC diet can cause inflammatory damage to the mammary gland tissue of dairy cows.

Autophagy is a cellular degradation process initiated in response to stress. It attempts to restore metabolic homeostasis through the catabolic lysis of aggregated proteins, which has important roles in cell self-renewal, maintenance of homeostasis, cell growth, and differentiation, which is a cytoprotective mechanism (29, 30). LC3 is a marker protein that can be used to detect autophagy, as it transforms from LC3I to activated LC3II during autophagy. p62 is an important transporter that acts as a bridge between LC3II and ubiquitinated substrates to be degraded (31, 32). Therefore, the content of p62 in cells can reflect the degree of autophagosome degradation. Beclin1 is a core gene regulating autophagy, which can enhance the intracellular Ca^2+^ signaling pathway by binding to inositol-1,4,5-triphosphate receptors (IP3Rs) thereby inducing autophagy (33–35). Therefore, Beclin1 is an indicator of autophagy activity. Previous studies have shown that overexpression of Beclin 1 can induce autophagy in MCF7 cells and HT29 cells (36). In the present study, the HC diet upregulated LC3 and Beclin1 at the gene and protein levels in mammary gland tissue of dairy cows, suggesting that the HC diet enhanced the autophagy activity of mammary gland tissue. The HC diet significantly reduced the expression of p62, increased the degree of autophagosome degradation, and upregulated the expression of autophagy-related proteins ATG5, ATG14, ATG16L, and LAMP2α. These results indicate that an HC diet induces autophagy in mammary gland tissue of dairy cows. *In vitro*, cell experiments showed that LPS stimulation significantly upregulated the expression of ATG5 and Beclin1 and decreased the protein expression of p62 in BMECs. Immunofluorescence results further indicated that LPS increased the fluorescence intensity of LC3 and decreased that of p62. These results suggest that LPS stimulation can induce autophagy in BMECs.

Elevated intracellular Ca^2+^ levels can induce autophagy by activating a variety of autophagy signaling kinases and proteasomes (37, 38). A study showed that AMPK could be activated by CaMKKβ when Ca^2+^ concentration was elevated in LKB1-deficient cells (39). Increased Ca^2+^ in the cytoplasm affects the activity of AMPK and mTOR through activation of CaMKKβ, thereby regulating key molecules of autophagy such as Beclin1 and ULK1 (40, 41). In a study of liver injury *in vitro* and *in vivo*, Singh and Kang found that Ca^2+^ release and expression of p-AMPK increased after FB1 treatment of HepG2 cells. Therefore, FB1-induced autophagy may be dependent on CaMKKβ-mediated activation of AMPK (42). Our results showed that an HC diet not only increased Ca^2+^ levels in mammary gland tissue of dairy cows but also significantly upregulated CaMKKβ expression. In addition, compared with an LC diet, the HC diet significantly increased the expression of AMPK and decreased the expression of mTOR in mammary gland tissue of dairy cows, suggesting that the HC diet may upregulate the expression of CaMKKβ, owing to the increased Ca^2+^ levels, and induce autophagy through the CaMKKβ/AMPK/mTOR signaling pathway. This was also verified *in vitro*: LPS stimulation increased calcium fluorescence intensity, upregulated CaMKKβ and AMPK, decreased the expression of mTOR protein, and increased the expression of autophagy-related genes and proteins.

In SARA state, rumen microbial cell bodies produce a variety of metabolites, such as LPS and γ-D-glutamyl-meso-diaminopimelic acid (iE-DAP) which lead to mammary gland damage (23, 43). At present, LPS is considered to be the most important bacterial endotoxin in the pathogenesis of SARA, and LPS is a common stimulator used to simulate SARA induction *in vitro* models. Our previous study found that LPS (2–20 μg/ml) could cause inflammatory damage to BMECs (Meng et al., 2022). First, we investigated the effects of LPS on autophagy of BMECs. We found that LPS induced autophagy in BMECs by upregulating the expression of autophagy-related proteins compared with the Ctrl group. Similarly, Ma et al. (44) showed that 4 μg/ml LPS stimulation induced autophagy and lipid metabolism disorders in BMECs. Next, we explored how LPS induced autophagy in BMECs. We found that compound C, an inhibitor of AMPK, could inhibit the phosphorylation of AMPKa induced by baicalin (45). First, we validated the inhibitory effect of compound C. The results showed that compound C pretreatment significantly inhibited the upregulation of AMPK protein in LPS-induced BMECs. Second, the BMECs were pretreated with compound C to study the effects of compound C on LPS-induced autophagy-related proteins. The results showed that compound C pretreatment significantly decreased Beclin1, ULK1, and ATG5 and increased the protein expression of p62. Compound C pretreatment also decreased the fluorescence intensity of LC3 and increased that of p62. These results suggest that AMPK signaling is involved in LPS-induced autophagy in BMECs.

CaMKKβ is one of the classical molecules upstream of AMPK, and a growing body of evidence confirms that it plays an important part in autophagy (18, 19). To determine whether CaMKKβ is involved in LPS-induced autophagy of BMECs, in the present study, we examined the effects of LPS on CaMKKβ expression. The results showed that LPS significantly upregulated CaMKKβ protein expression. STO-609 is an inhibitor of CaMKKβ. Then, we pretreated BMECs with STO-609 before treating them with LPS and detecting the expression of CaMKKβ and autophagy-related genes and proteins. The results showed that STO-609 pretreatment could significantly reduce the expression of CaMKKβ, which confirmed the inhibitory effect of STO-609, consistent with the results of Fujiwara et al. (46). Research has shown that pretreatment with STO-609 (an inhibitor of CaMKKβ) not only inhibited AMPK phosphorylation but also inhibited Ca^2+^-mediated autophagy (47, 48). This was consistent with our findings. Our study found that STO-609 pretreatment reduced ULK1, Beclin1, and LC3 expression, and elevated p62 protein expression. In addition, immunofluorescence results showed that STO-609 pretreatment reduced the fluorescence intensity of LC3 and increased the fluorescence intensity of p62. This suggests that inhibition of CaMKKβ can alleviate LPS-induced autophagy. These results suggest that CaMKKβ plays an important part in the LPS-induced autophagy pathway in BMECs. Studies have shown that sodium butyrate activates the CaMKKβ pathway to mediate AMPK phosphorylation (49), and that STO-609 pretreatment significantly inhibits apigenin-induced AMPK phosphorylation (50). To further confirm the important role of AMPK in the LPS-induced autophagy pathway in BMECs, we pretreated BMECs with STO-609 and then stimulated them with LPS to investigate the effects of CaMKKβ inhibition on the AMPK signaling pathway. STO-609 pretreatment significantly inhibited the protein expression of AMPK in LPS-induced BMECs. These results suggest that AMPK signaling is involved in LPS-induced autophagy in BMECs. In conclusion, the CaMKKβ–AMPK signaling pathway is involved in LPS-induced autophagy of BMECs.

## 5 Conclusions

**Figure 7:**
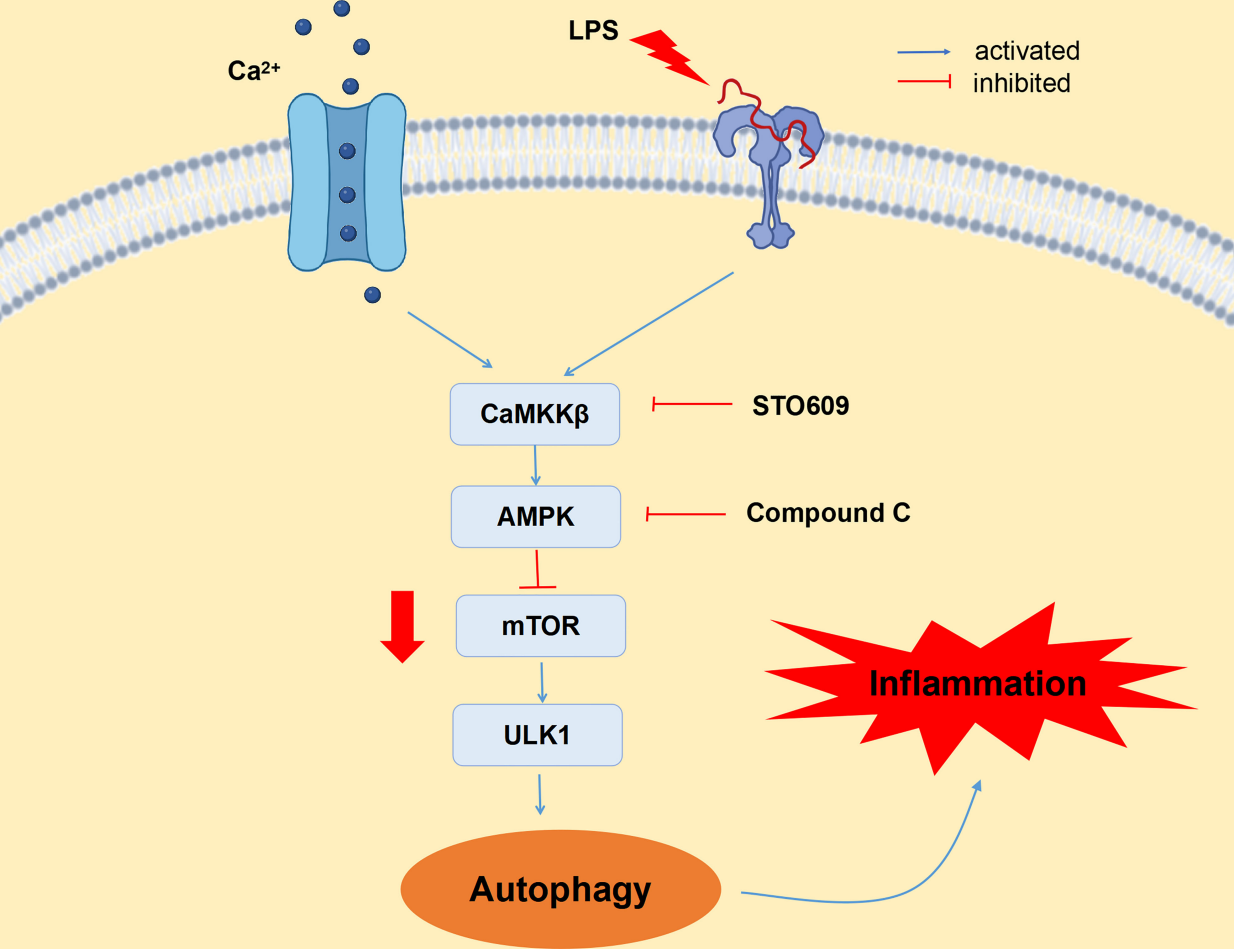
CaMKKβ–AMPK signaling pathway is involved in LPS-induced autophagy in bovine mammary epithelial cells. LPS upregulated CaMKKβ expression by increasing intracellular calcium ion content, thereby activating AMPK signaling pathway and thus inducing autophagy. LPS induces BMECs inflammatory injury by activating autophagy through the CaMKKβ–AMPK signaling pathway.

An HC diet induces SARA by decreasing rumen pH and increasing LPS content in blood, which results in inflammatory damage to mammary gland tissue of dairy cows. In addition, feeding an HC diet can activate CaMKKβ by increasing the content of Ca^2+^ in mammary gland tissue of dairy cows, which then mediates the activation of AMPK and reduces the expression of mTOR, inducing autophagy through the CaMKKβ/AMPK/mTOR signaling pathway. Moreover, inhibition of CaMKKβ and AMPK reduces LPS-induced autophagy, thereby alleviating inflammatory injury of BMECs. Thus, CaMKKβ–AMPK signaling is involved in LPS-induced autophagy in BMECs (**Figure 7**). Therefore, CaMKKβ can be used as a clinical target for the treatment and prevention of dairy cow mastitis.

## Data availability statement

The original contributions presented in the study are included in the article/Supplementary Material. Further inquiries can be directed to the corresponding author.

## Ethics statement

The experimental procedures were approved in accordance with the Guidelines for Experimental Animals of the Ministry of Science and Technology (2006, Beijing, China) and were allowed by the Animal Ethics Committee of Nanjing Agricultural University with approval of SYXK-2017-0027.

## Author contributions

MM and XS conceived and designed the experiment. MM was responsible for the implementation of experiments, and the writing, review, and revision of papers. XL, ZW, RH, NM, and GC provided experimental operation and statistical analysis. XS and GC provided technical and material support. All authors contributed to the article and approved the submitted version.
