# Supplementary figures and images for: A high-concentrate diet induces inflammatory injury via regulating Ca2+/CaMKKβ-mediated autophagy in mammary gland tissue of dairy cows
Source: Front Immunol. 2023 May 1;14:1186170. doi: 10.3389/fimmu.2023.1186170 (PMC10183583; doi:10.3389/fimmu.2023.1186170)

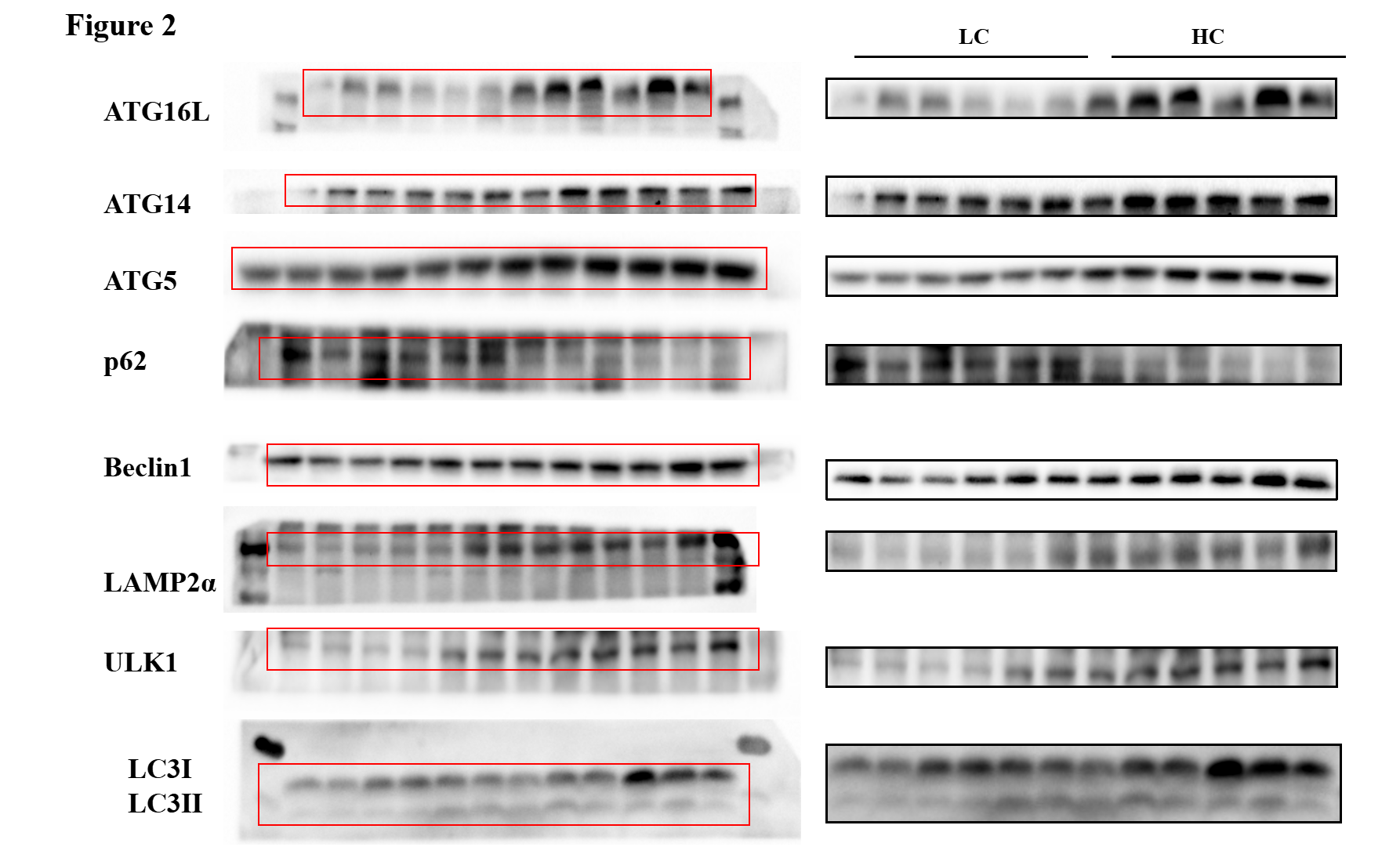


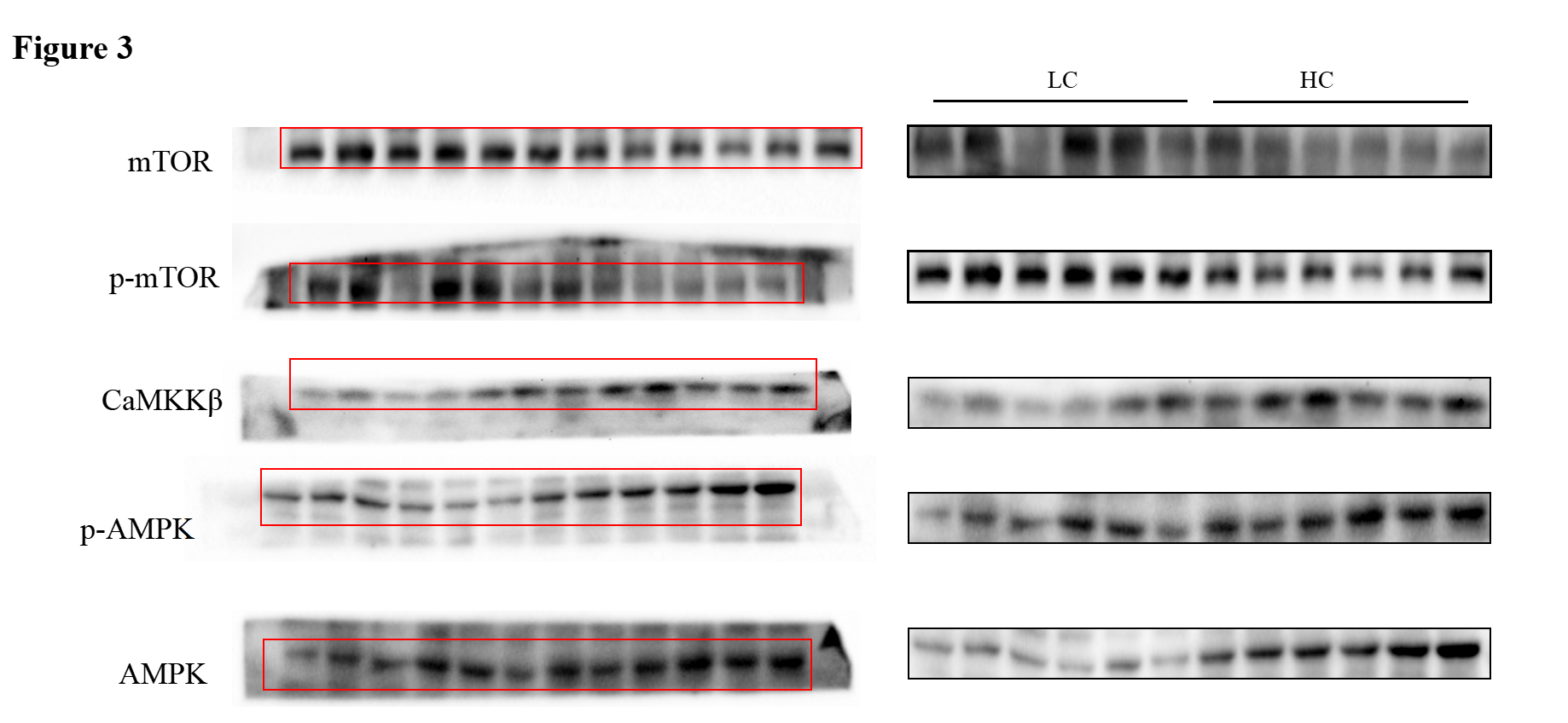


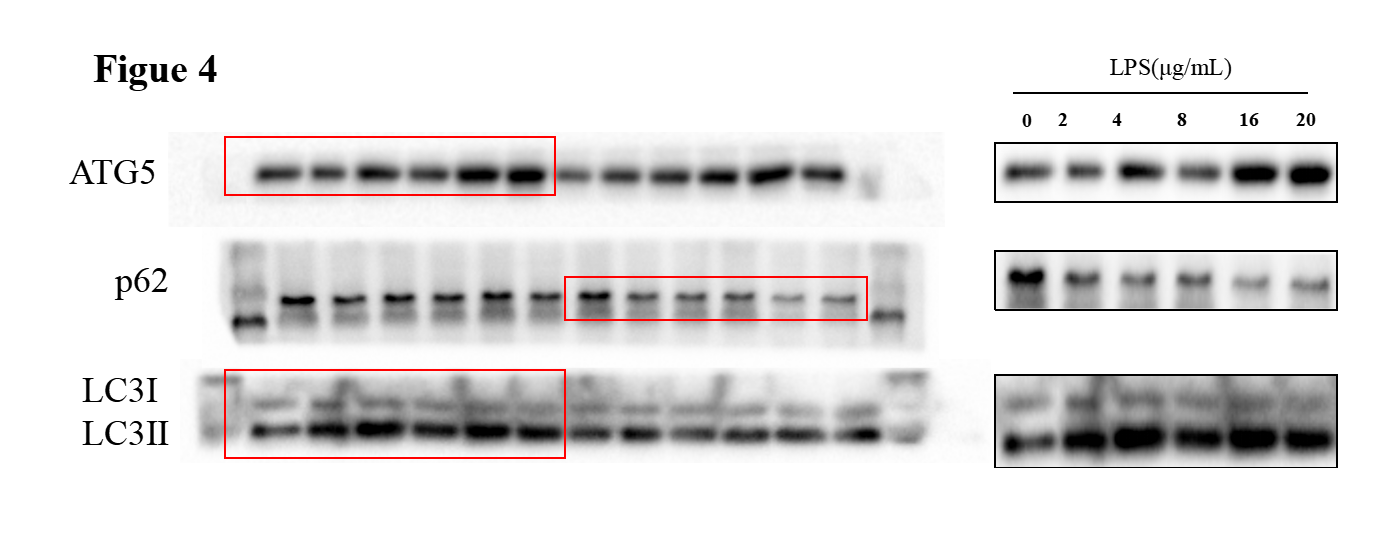


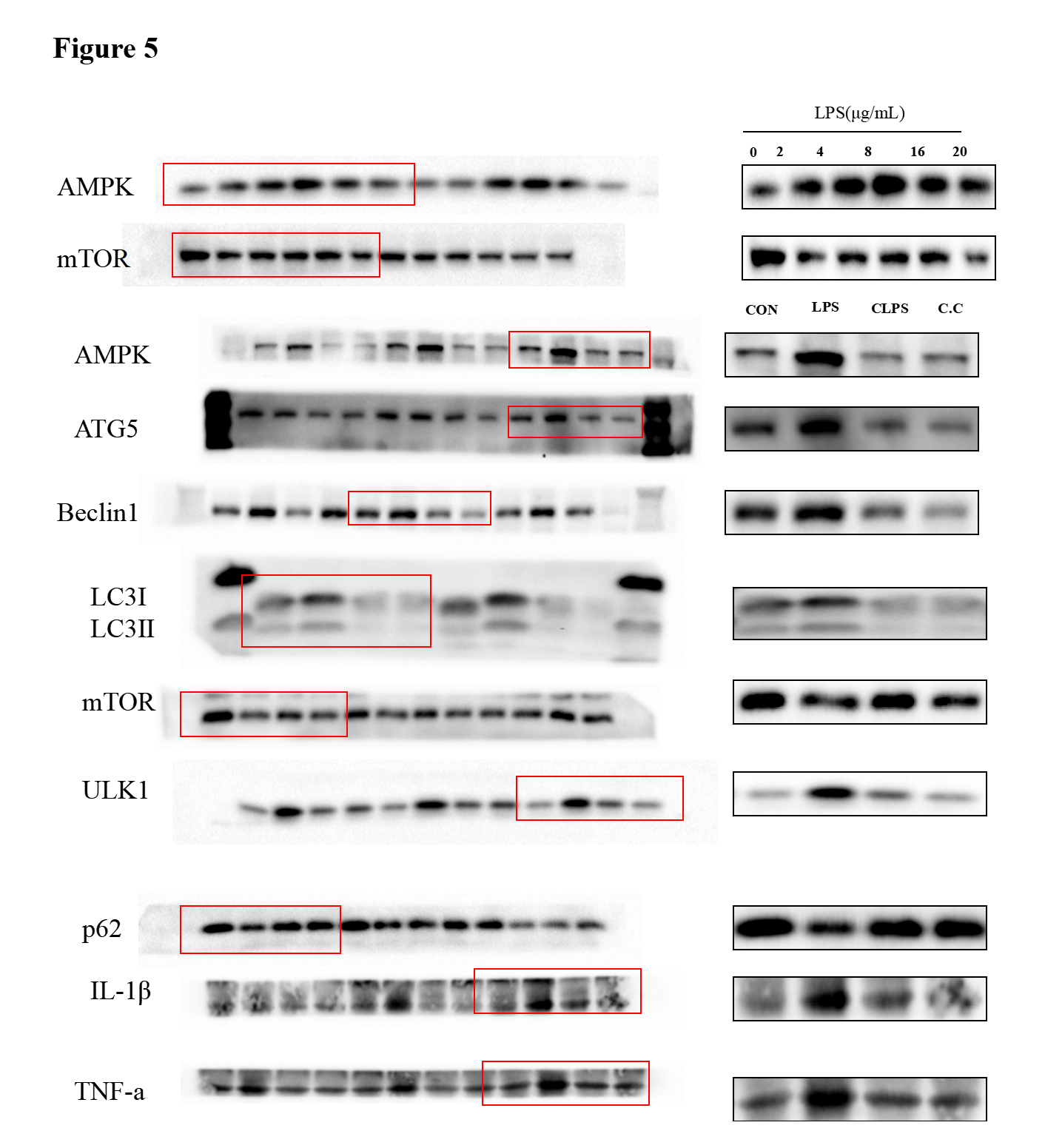


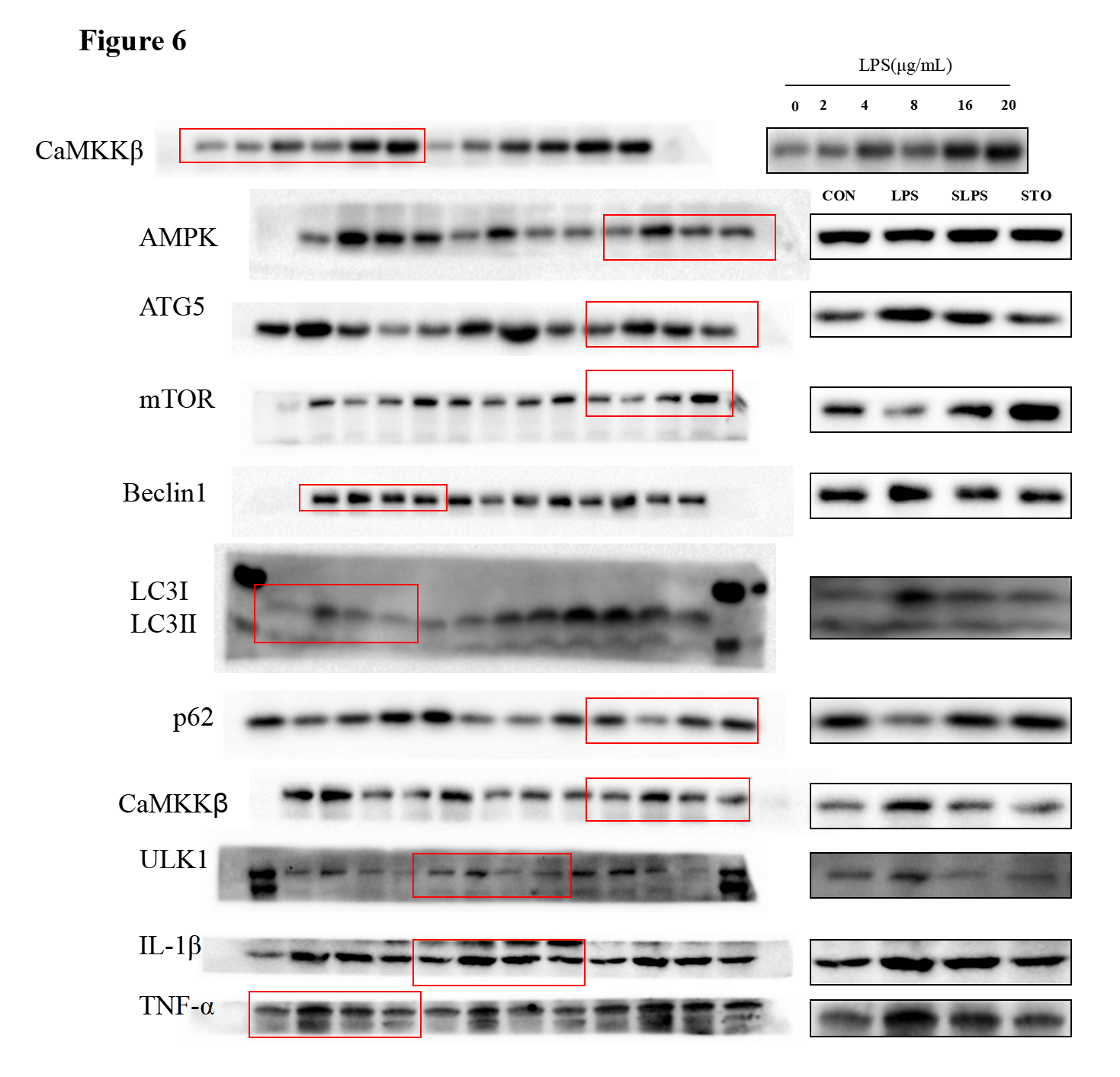


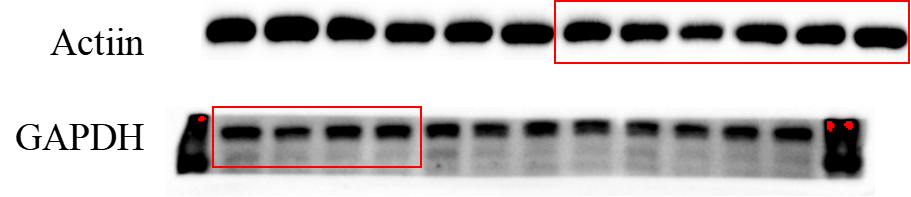

Supplement: Supplementary file 1 [file DataSheet_1.docx]
